# No difference in long-term functional outcomes or survivorship after total knee arthroplasty with or without computer navigation: a 17-year survivorship analysis

**DOI:** 10.1186/s43019-021-00114-2

**Published:** 2021-09-08

**Authors:** Ng Jonathan Patrick, Lau Lawrence Chun Man, Chau Wai-Wang, Ong Michael Tim-Yun, Cheung Kin Wing, Chiu Kwok Hing, Chung Kwong Yin, Ho Kevin Ki-Wai

**Affiliations:** 1 Department of Orthopaedics and Traumatology, Prince of Wales Hospital, Shatin, Hong Kong SAR China; 2 Department of Orthopaedics and Traumatology, Chinese University of Hong Kong, Shatin, Hong Kong SAR China

**Keywords:** Computer-assisted navigation surgery, Total knee replacement, Outcomes, Survivorship

## Abstract

**Background:**

The literature comparing the long-term outcomes and survivorship of computer navigation-assisted and conventional total knee replacement (TKR) is sparse. Moreover, of the available comparative studies with follow-up duration of more than 10 years, the results seem to be conflicting. The purpose of this long-term study was to compare the clinical and radiological outcomes, and implant survivorship, of TKR performed with and without computer navigation.

**Methods:**

We retrospectively compared the results of 49 computer-navigated TKRs and 139 conventional TKRs. The mean age of the patients was 67.9 (range 52–81) years for the navigation group and 67.1 (range 50–80) years for the conventional TKR group. The mean duration of follow-up for the conventional and navigation TKR groups was 12.9 and 13.2 years, respectively. Clinical and radiographic follow-up examinations of the patients were performed at 2 weeks, 1 month, 3 months and 6 months post-operatively, and at 1-year intervals thereafter.

**Results:**

There were no significant differences in the post-operative Knee Society knee and function score between the two groups. The mean overall deviation from neutral alignment and the radiological outliers were significantly higher in the conventional TKR group. The overall survival rates at 17 years were 92.9% for the navigation group and 95.6% for the conventional TKR group (*p* = 0.62).

**Conclusions:**

Navigated TKR resulted in fewer radiological outliers; however, this did not translate to better long-term functional outcomes or implant survival.

## Background

Computer navigation-assisted total knee replacement (TKR) has been in clinical use since the early 2000s. The technology was first introduced in an effort to reduce implant malalignment and, in doing so, to improve functional outcomes and implant survival. Indeed, some studies have shown more accurate radiographic alignment achieved with navigation-assisted TKR [1–6]. However, non-supporters cite increased costs, extended operative times, pin-site complications and system failures as the drawbacks of this technology [4–6]. To date, the majority of comparative studies in the literature evaluating the outcomes of conventional and computer-navigated TKR have mainly investigated short-to-mid-term outcomes, with only a handful of studies reporting long-term outcomes of these patients [7–9]. Furthermore, the evidence on whether improved alignment and position of the components improved longevity of the TKR and function has been inconclusive [10]. De Steiger et al. examined the Australian National Joint Registry data and compared the cumulative revision rate of 44,473 navigation-assisted TKR versus 270,545 conventional TKR over 9 years of follow-up duration. Based on their analysis, they concluded that, for the subgroup of patients < 65 years old, computer-assisted navigation TKR resulted in a significant reduction in revision for aseptic loosening with a hazard ratio of 1.38 [11]. In contrast, in one of the longest follow-up studies to date, Kim et al.’s randomised controlled trial (RCT) found no difference in clinical and radiological outcomes, and survivorship, in navigation-assisted TKR compared with conventional TKR [9]. Interestingly, in the studies performed by De Steiger et al. and Kim et al., the follow-up durations were 9 and 12 years, respectively. However, previous studies demonstrated that malaligned TKRs failed more often after 10 years of follow-up [11–13]. Therefore, whether an improvement in component alignment achieved with computer-assisted navigation translated to lower revisions rates and implant longevity may only become apparent with a significantly longer follow-up duration. In light of this, this study aimed to compare the long-term clinical and radiographic outcomes, and the implant survivorship, of navigation-assisted TKR with conventional TKR. In addition, we also assessed whether complication rates would be lower for computer-navigation TKR compared with conventional TKR. We hypothesised that the radiographic outcomes and consequent implant survivorship may be better in the navigation-assisted TKR group.

## Methods

A total of 188 patients were evaluated for this study. Female patients constituted 83.0% of the overall treatment groups, and the mean age at the time of surgery for the computer navigation (NAV-TKR) and conventional surgery (CONV-TKR) groups was 67.9 (range 52–81) and 67.1 (range 50–80) years old, respectively. Forty-nine primary TKRs were performed with image-less infrared computer navigation system in our institution between November 2002 and December 2005. Two computer tomography free navigation systems, VectorVision (Brainlab AG, Feldkirchen, Germany) and Stryker Navigation 2.0 (Stryker Mahwah, NJ), were used. The results were compared with 139 consecutive primary TKRs performed with conventional technique within the same period. Patients were assigned to have CONV-TKR or NAV-TKR depending on the availability of instruments for navigation surgery. No other selection criteria were used to assign the patients to either CONV-TKR or NAV-TKR. Ethics approval was obtained from a local research ethics committee (approval no. 2020.235). Inclusion criterium was end-stage osteoarthritis of the knee that failed conservative management. Patients with previous fracture, knee infection, or surgeries were excluded from the study. No patients were lost to follow-up.

All TKRs were either performed or supervised by a specialist arthroplasty surgeon with at least 10 years experience in adult joint replacement. Cemented posterior stabilised prostheses were implanted in all TKRs via medial parapatellar approach. Intra-medullary femoral guide and extra-medullary tibial guide were used in the TKRs performed with the conventional technique. Three brands of prosthesis were used: Scorpio PS (Osteonics, Allendale, NJ, USA), PFC Sigma (DePuy, Warsaw, IN, USA) and Nex-Gen Legacy (Zimmer, Warsaw, IN, USA) were implanted. All three TKR systems used were posterior substituting implants.

Patients were followed up at 2 weeks, 1 month, 3 months and 6 months post-operatively, and at 1-year intervals thereafter. The mean duration of follow-up was 13 (range 2.27–17.37) years. The Knee Society knee score and function score was recorded at each visit [14]. A standard goniometer was used to measure the range of knee motion pre-operatively and at each post-operative visit. Furthermore, radiographic assessment was also done at each post-operative visit. The overall lower limb alignment (measured by the mechanical tibiofemoral angle), the position of the components and any presence of radiolucent lines surrounding the implants were assessed on anteroposterior lower-limb scanogram, as well as the standing anteroposterior, lateral and skyline patellar radiographs. The radiographic alignment in both the coronal and sagittal planes were compared using the Knee Society roentgenographic evaluation and scoring system [15]. The degree of deviation from neutral alignment was compared in the femoral coronal plane, the tibial coronal plane, the overall coronal plane, the femoral sagittal plane and the tibial sagittal plane. Absolute degree of deviation from neutral alignment was used for analysis, as deviation in either direction should not cancel each other out, resulting in underestimation of the actual deviation. Deviation of more than 3° from neutral alignment was defined as an outlier.

### Statistical analysis

Statistical analysis was performed using IBM SPSS version 27 (Armonk, NY: IBM Corp). Demographic statistics on age, sex, body mass index and mean pre-operative absolute deformity were reported in terms of mean ± SD or ratio where appropriate. Kaplan–Meier survival analysis was performed to determine the mean survival rates and survival years of the implants, with 95% confidence intervals (CIs). Statistical significance was calculated through log rank test. The endpoint for survivorship was defined as revision TKR for any cause. Revision TKR included any knee exploration following TKR including exchange of insert, tibial or femoral component for aseptic or septic reasons, including component malalignment, osteolysis and component failure. Pre-operative and post-operative clinical data, including the range of motion of the knee using Mann–Whitney *U* test, and Knee Society knee score and function score were compared using Student’s *t*-test. The level of significance was set at *p* < 0.05.

## Results

A total of 188 knees were recruited in this study, 49 TKRs (26.1%) performed with navigation assistance and 139 TKRs (73.9%) performed with the conventional technique. The longitudinal mean follow-up visit for the NAV-TKR group was 13.19 (range 2.75–17.20) years, and 12.90 (range 2.27–17.37) years for the CONV-TKR group. The baseline demographic data and pre-operative clinical and radiographic data are presented in Tables 1 and 2a. The primary diagnosis was osteoarthritis in 93.8% of patients. The mean duration of surgery for CONV-TKR and NAV-TKR was 87.70 (range 53–144) and 121.98 (range 89–169) min, respectively (*p* < 0.01).

**Table 1:** Baseline characteristics of all recruited patients

	Mean (range)		*p*-Value
	NAV-TKR (*N* = 49)	CONV-TKR (*N* = 139)	
Age (years)	67.90 (52–81)	67.06 (50–80)	0.51
Sex distribution	10:39	22:117	0.51
Body mass index	29.7 (16.5–45.8)	28.9 (18.6–44.8)	0.26
Diagnosis	47 OA: 2 RA	133 OA: 6 RA	0.74
Mean pre-operative absolute deformity	10.8° (15° valgus to 20° varus)	10.68° (20° valgus to 20° varus)	0.83

**Table 2:** Comparing the range of motion, Knee Society knee score and Knee Society function score by (a) groups, (b) time points

(a) Comparing NAV-TKR and CONV-TKR groups			
	NAV-TKR	CONV-TKR	*p*-Value
*Pre-operative*			
Range of motion (degrees)	92.35 ± 19.10	94.86 ± 17.78	0.42
Knee Society knee score	28.84 ± 13.35	32.35 ± 15.75	0.17
Knee Society function score	52.18 ± 11.34	50.47 ± 16.18	0.91
*Post-operative*			
Range of motion (degrees)	98.47 ± 13.63	103.66 ± 13.62	0.02
Knee Society knee score	90.57 ± 12.18	89.95 ± 15.75	0.80
Knee Society function score	59.15 ± 22.04	55.48 ± 29.49	0.21

(b) Comparing pre-operative and post-operative scores			
	Pre-operative	Post-operative	*p*-Value
*NAV-TKR*			
Range of motion (degrees)	92.35 ± 19.10	98.47 ± 13.63	0.02
Knee Society knee score	28.84 ± 13.35	90.57 ± 12.18	< 0.01
Knee Society function score	52.18 ± 11.34	59.15 ± 22.04	0.04
*CONV-TKR*			
Range of motion (degrees)	94.86 ± 17.78	103.66 ± 13.62	< 0.01
Knee Society knee score	32.35 ± 15.75	89.95 ± 15.75	< 0.01
Knee Society function score	50.47 ± 16.18	55.48 ± 29.49	0.04

Mean ± SD

*SD* standard deviation

For the clinical outcomes, the range of motion and Knee Society knee and function scores improved significantly compared with pre-operative scores in both groups (Table 2b). Furthermore, there were no significant differences in the post-operative Knee Society knee and function scores between the two groups (Table 2a). Interestingly, the mean post-operative range of motion appeared to be significantly better in the CONV-TKR group (98.47° versus 103.66°, *p* = 0.02).

In terms of alignment, the mean pre-operative absolute deformity for NAV-TKR and CONV-TKR was 10.8° (range 15° valgus to 20° varus) and 10.68° (range 20° valgus to 20° varus), respectively (*p* = 0.83). Post-operatively, the mean overall deviation from the neutral alignment in the coronal plane (i.e. mechanical tibiofemoral angle of zero degrees) was 1.89° (range 0–7) for the NAV-TKR group, and 2.62° (0–9.5) for the CONV-TKR group (*p* = 0.01) (Table 3). Similarly, the radiological outliers for the femoral and tibial components, defined as less than 3° from neutral, were significantly higher in the CONV-TKR group (Table 3).

**Table 3:** Comparing the deviations in different planes from neutral alignment and identification of outliers between NAV-TKR and CONV-TKR groups

	Mean (range)		*p*-Value
	NAV-TKR	CONV-TKR	
*Deviation in different planes from neutral alignment (degrees)*			
Femoral coronal plane	1.00 (0–5)	1.53 (0–10)	0.01
Tibial coronal plane	1.24 (0–5)	1.91 (0–7.5)	< 0.01
Overall coronal plane	1.89 (0–7)	2.62 (0–9.5)	0.01
Femoral sagittal plane	2.04 (0–9)	3.27 (0–10)	< 0.01
Tibial sagittal plane	1.99 (0–10)	2.55 (0–14)	0.04
*Outliers (> 3°)*			
Femoral coronal plane	0 (0%)	18 (12.5%)	0.01
Tibial coronal plane	3 (6.1%)	29 (20.1%)	0.03
Overall coronal plane	9 (18.3%)	46 (31.9%)	0.01
Femoral sagittal plane	10 (20.4%)	70 (48.6%)	< 0.01
Tibial sagittal plane	16 (32.7%)	48 (33.3%)	1.00

Nine revision cases were recorded in total in this study. In the NAV-TKR group, there was one infection (2.04%) (at 3 years) and two aseptic loosenings (4.08%) (at 10.4 and 11.1 years), leading to revision surgery. In the CONV-TKR group, there were four cases of infection (2.88%) (at 0.9, 1.91, 1.94 and 2.2 years) and two aseptic loosenings (1.44%) (at 6.9 and 7.4 years), leading to revision surgery. In addition, there were no significant differences in terms of other complications, including superficial wound infection (NAV-TKR 6.12% versus CONV-TKR 4.86%, *p* = 0.72), deep vein thrombosis (NAV-TKR 4.08% versus CONV-TKR 4.86%, *p* = 1.00) or post-operative stiffness requiring manipulation under anaesthesia (NAV-TKR 8.16% versus CONV-TKR 9.03%, *p* = 1.00).

**Fig. 1:**
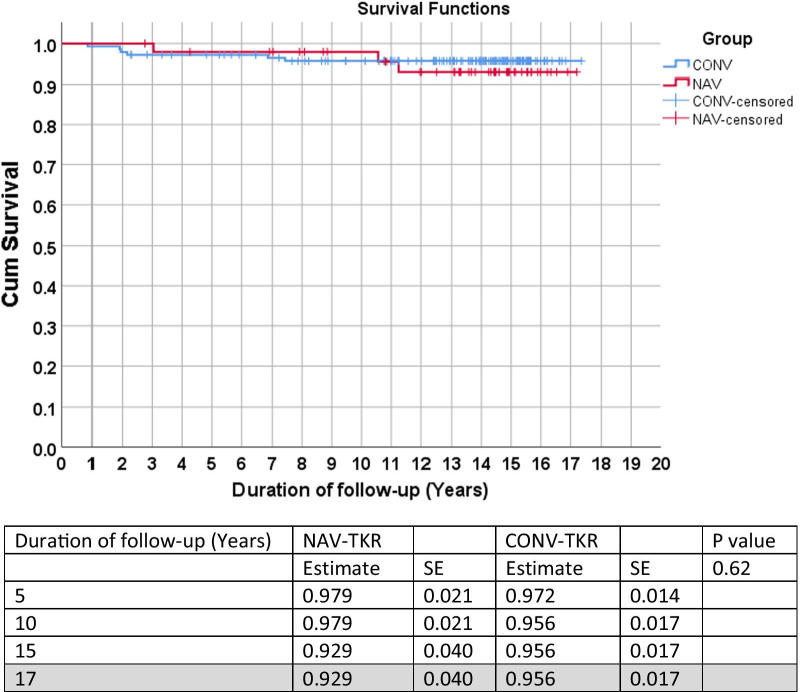
All-cause revision surgery for survival estimates as an endpoint. *SE* standard error

**Fig. 2:**
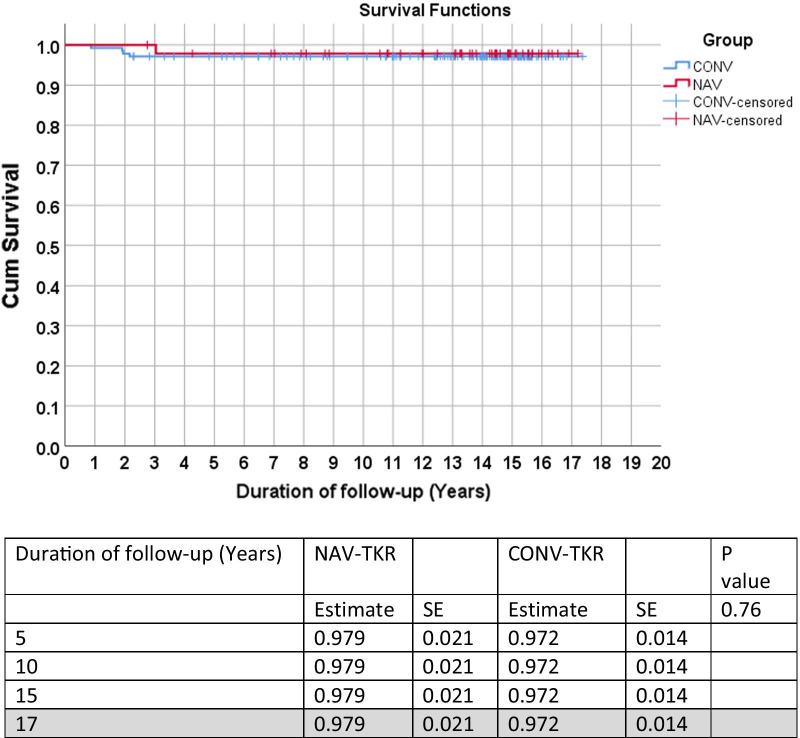
Infection cause of revision surgery on estimate for survival endpoint. *SE* standard error

**Fig. 3:**
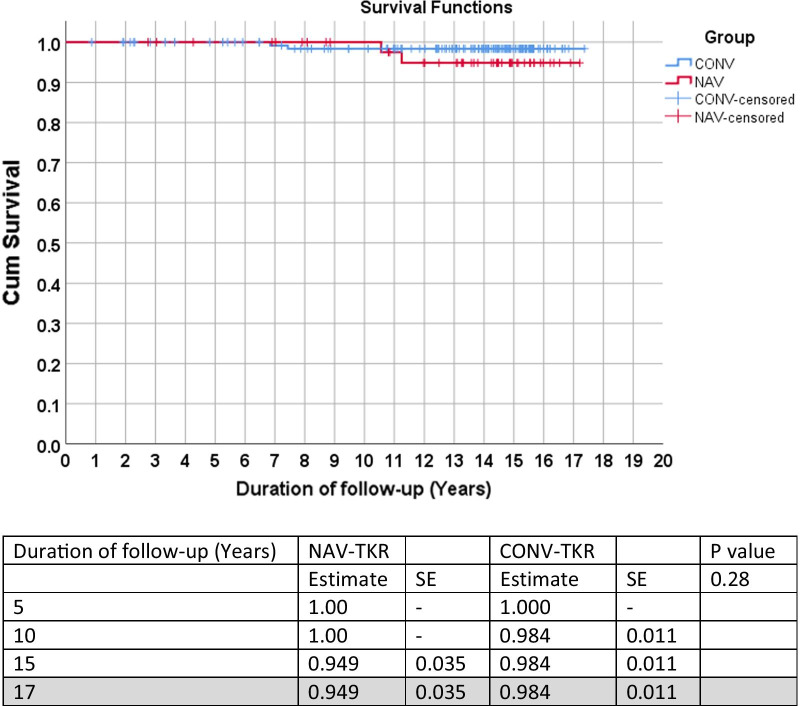
Revision causes of aseptic loosening on estimate as survival endpoint. *SE* standard error

**Fig. 4:**
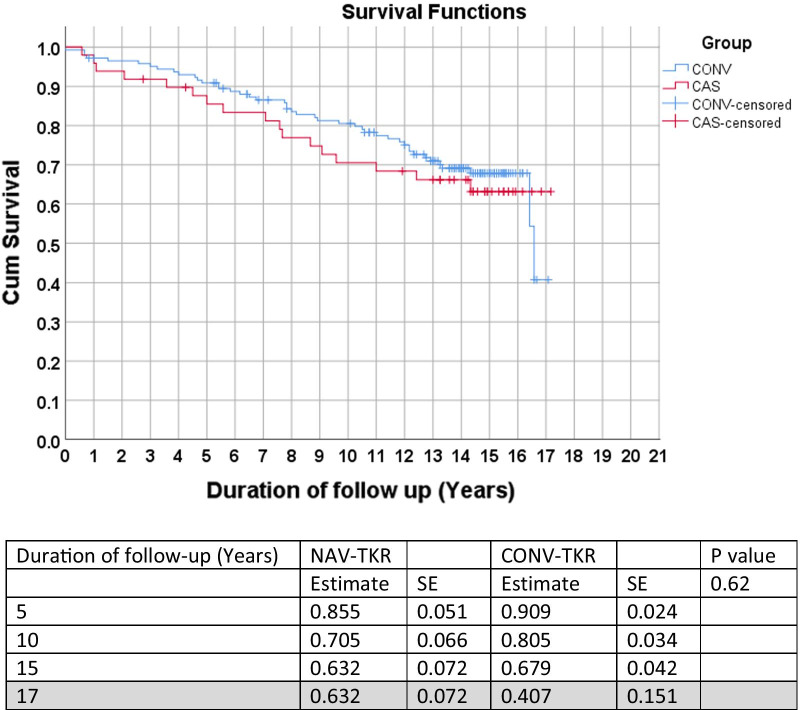
Kaplan–Meier survival estimate of death as an endpoint. *SE* standard error

Using revision surgery for all causes as the endpoint, there was no statistically significant difference between the two groups across all time points (*p* = 0.62) (Fig. 1). Similarly, no statistical difference was noted between the two groups when revision for infection or aseptic loosening were used as the endpoints (Figs. 2 and 3). Lastly, no statistical difference between the two groups was found when death was the endpoint of censorship (Fig. 4).

The overall survival estimates for revision surgery for all causes were 16.76 (95% CI 16.36, 17.16) years, of which mean was 16.59 (95% CI 15.89, 17.29) years for the NAV-TKR group and 16.77 (95% CI 16.29, 17.24) years for the CONV-TKR group (Fig. 1). Furthermore, when endpoints were censored by revisions for aseptic loosening, the overall survival estimate was 17.16 (95% CI 16.96, 17.36) years for NAV-TKR and 17.20 (95% CI 16.98, 17.43) years for CONV-TKR (Fig. 3).

Lastly, there was no significant difference in implant survivorship between the three brands of TKR used in this study.

## Discussion

The most important finding from this study was that navigation-assisted TKR resulted in fewer radiological outliers; however, this did not translate to better long-term functional outcomes or implant survival. To the author’s knowledge, of the available comparative studies on computer navigation versus conventional TKR, our study has the longest duration of follow-up.

Numerous studies have evaluated whether computer-navigation TKR resulted in more accurate component alignment. Navigated TKR was first introduced for the purpose of obtaining better alignment, based on the assumption that restoring neutral mechanical alignment could maximise implant survival. Encouragingly, several studies have found that computer-navigated total knee arthroplasty achieved better outcomes in post-operative alignment [16–21]. However, these results were contradicted equally by similar studies that showed no superiority in achieving accurate alignment with the use of navigation systems [22–24]. Based on our study, we were able to achieve a better neutral mechanical alignment and fewer component outliers with navigation. Our results were consistent with several meta-analyses which also suggested that navigated TKR resulted in a significant improvement in component orientation and restoration of the mechanical axis compared with the conventional technique [19, 21, 25–27].

Nevertheless, whether the difference in the accuracy of component alignment is a critical factor that affected long-term outcomes has not been fully elucidated, with conflicting evidence in the literature. Baumbach et al. [8] compared the 10-year survival of 50 navigated and 46 conventional TKRs and found that 17% of the knees that had conventional TKR and 9.8% of those that had computer-navigated TKR had aseptic loosening. All of the knees that had aseptic loosening had a mechanical tibiofemoral angle outside the recommended range of ± 3°. Based on their results, the authors concluded that their study demonstrated a clinically important advantage of the computer-navigation technique compared with the conventional procedure. Conversely, Kim et al.’s prospective, randomised controlled trial with a mean follow-up of 12.3 years demonstrated similar clinical function and survivorship of the components between the navigation and conventional surgery groups. The authors reported implant survivorship, with revision or aseptic loosening defined as the endpoint, of 100% for both groups at 13 years follow-up [9]. At 17 years of follow up, our study echoed these findings, demonstrating a similar survivorship between between the navigation and conventional surgery groups of 92.9% and 95.6%, respectively.

Recently, a meta-analysis of nine randomised controlled trials which included 1348 computer-navigated and 1347 conventional TKRs, with a mean follow-up ranging from 9 to 15 years, demonstrated that computer-navigated TKR resulted in better outcomes in post-operative component alignment [28]. However, there were no significant differences in long-term functional outcomes and survivorship between the two techniques. The results from our study were in line with the findings from this meta-analysis. However, it is important to note that both the conventional and navigated TKRs in the RCTs included in the meta-analysis were often performed by the senior authors with extensive experience in arthroplasty, in a better-than-average surgical environment. Hence, any differences in the clinical and radiological outcomes between the two techniques may not be as apparent. In contrast, in our study, although all TKRs were supervised by a specialist arthroplasty surgeon with at least 10 years experience, the execution of the registration and bone cuts were performed by surgeons with variable experiences, ranging from residents to consultants. Despite this, the radiological outcomes still seemed to be superior in the navigation group, highlighting the advantages of navigation in allowing low-volume or less experienced surgeons to achieve similar surgical accuracy compared with high-volume surgeons. Furthermore, the fact that the results from our study may be generalised across the entire spectrum of surgeons with different levels of skills may be one of the strengths of our study.

The Australian Orthopaedic Association National Joint Replacement Registry (AOANJRR), which has listed 153,056 recorded primary computer-navigated TKRs in its current rendition, suggested navigated TKR showed a significant reduction in revision secondary to component loosening (0.8% less for < 65 years old, 0.3% less for ≥ 65 years old) in all age groups at 15 years of follow-up [29]. Compared with the Australian registry, which reported that computer navigation was used in 33.3% of all primary TKR in 2019, the proportion of navigated TKR is much less in our locality, with figures likely similar to the UK (3%) [30] or the USA (7%) [31]. Therefore, the supposed superior long-term outcomes of navigated TKR shown in the Australian registry may not be translatable to other populations. For instance, the American Academy of Orthopaedic Surgeons in 2016 made a strong recommendation stating that the evidence to date supported not using computer navigation in TKR as there was no difference in outcomes or complications. Therefore, despite different “level 1” or “big data” evidence available, there is still no consensus on this debate. In spite of the relatively small sample size in our study, our results add to the current body of evidence regarding navigation TKR in the local population.

Lastly, the reason for the poorer flexion range in the navigation group (mean 94.83°) than in the conventional group (mean 103.82°) is not known. We postulated that it may be the result of increased quadriceps scarring and adhesions from the femoral pin tracks. Further studies are warranted to investigate whether this may be mitigated with the use of image-less navigation systems where tracker pins are not needed.

Our study has several limitations. Firstly, this was a retrospective cohort study with a relatively small sample size, despite the long duration of follow-up. Secondly, two different navigation systems and three different implants were used in this study, although the results were quite consistent, which may also suggest that accurate alignment and reduction of outliers could be achieved irrespectively of the brand of the navigation system. Thirdly, our series was limited to the southern Chinese population, with few obese and no morbidly obese patients. Therefore, the benefit of computer navigation in identifying normal anatomic landmark in otherwise obese patients may not be as obvious in our series. Similarly, the advantages of using navigation in TKR in more complex cases such as extra-articular deformity [32, 33], retained hardware (e.g. previous high tibial osteotomy), presence of ipsilateral total hip replacement and ankylosed knees were also not explored in our study. The benefits of navigation may become more apparent in these complex cases where conventional jigs relying on relatively normal anatomy are less accurate. The difference in the sample size between the NAV-TKR group and CONV-TKR group exerts an effect on the statistical power of this study, which limits the data generalisability. Lastly, due to the small number of revisions, the association between component alignment and survivorship could not be determined.

## Conclusion

In the present study, we showed that navigated TKR resulted in fewer radiological outliers; however, this did not translate to better long-term functional outcomes or implant survival. The current evidence on whether improved alignment and position of the components achieved with navigation TKR improved longevity of the TKR and function remains conflicting.

## Data Availability

The datasets used and/or analysed during the current study are available from the corresponding author on reasonable request.

## Abbreviations

TKR: Total knee replacement
RCT: Randomised controlled trial
NAV-TKR: Computer navigation-assisted total knee replacement
CONV-TKR: Conventional total knee replacement
SD: Standard deviation
CI: Confidence interval
AOANJRR: Australian Orthopaedic Association National Joint Replacement Registry
